# Local Anesthesia

**Published:** 1904-06

**Authors:** 


### Local Anesthesia.

Horatio C. Wood, of Philadelphia, (*Therapeutic Review,* Feb., 1904,) says the suggestion of Barker, that eucain be substituted for cocain, seems to have brought infiltration anesthesia almost to the high water mark and has been followed out in Philadelphia by various surgeons with great satisfaction. The plan is readily carried out as follows: powders are kept in stock in the operating room containing 0.02 grm. (3 grains) of beta-eucain and 0.8 grm. (12 grains) of pure sodium chloride. At the time of the operation one such powder is dissolved in 100 cc. of boiling distilled water, and when it is cooled sufficiently 1 cc. of adrenalin chloride solution (1-1,000) is added; 100 cc. of the resulting liquid contains 3 grains of B-eucain and 0.015 grains of adrenalin chloride. The whole 100 cc. may be used at one infiltration anesthesia, but according to Barker from 50 to 60 cc. usually suffices, even in such considerable operations as for the cure of hernia, castration and the like. It is of course essential that the syringe used be aseptic and the surgeon should always employ the platinum needle with an iridium point which can, without injury, be disinfected in the flame of an alcohol lamp immediately before use.

